# Dental and Oral Manifestations of COVID-19 Related Mucormycosis: Diagnoses, Management Strategies and Outcomes

**DOI:** 10.3390/jof8010044

**Published:** 2021-12-31

**Authors:** Omer Sefvan Janjua, Muhammad Saad Shaikh, Muhammad Amber Fareed, Sana Mehmood Qureshi, Muhammad Ikram Khan, Danya Hashem, Muhammad Sohail Zafar

**Affiliations:** 1 Department of Maxillofacial Surgery, PMC Dental Institute, Faisalabad Medical University, Faisalabad 38000, Pakistan; osj1982@hotmail.com; 2 Department of Oral Biology, Sindh Institute of Oral Health Sciences, Jinnah Sindh Medical University, Karachi 75510, Pakistan; drsaadtanvir@gmail.com; 3 Adult Restorative Dentistry, Dental Biomaterials Science and Prosthodontics Oman Dental College, Muscat 116, Oman; mafareed@staff.omandentalcollege.org; 4 Department of Oral Pathology, PMC Dental Institute, Faisalabad Medical University, Faisalabad 38000, Pakistan; sana.mehmood@outlook.com; 5 Department of Oral and Maxillofacial Surgery, King Edward Medical University, Lahore 54000, Pakistan; drikramazad@gmail.com; 6 Department of Restorative Dentistry, College of Dentistry, Taibah University, Al Madinah, Al Munawwarah 41311, Saudi Arabia; dhashem@taibahu.edu.sa; 7 Department of Dental Materials, Islamic International Dental College, Riphah International University, Islamabad 44000, Pakistan

**Keywords:** aspergillosis, SARS-CoV-2, mucormycosis, fungal infection, oral mucormycosis

## Abstract

It has been nearly two years since the pandemic caused by the novel coronavirus disease (COVID-19) has affected the world. Several innovations and discoveries related to COVID-19 are surfacing every day and new problems associated with the COVID-19 virus are also coming to light. A similar situation is with the emergence of deep invasive fungal infections associated with severe acute respiratory syndrome 2 (SARS-CoV-2). Recent literature reported the cases of pulmonary and rhino-cerebral fungal infections appearing in patients previously infected by COVID-19. Histopathological analysis of these cases has shown that most of such infections are diagnosed as mucormycosis or aspergillosis. Rhino-orbital-cerebral mucormycosis usually affects the maxillary sinus with involvement of maxillary teeth, orbits, and ethmoidal sinuses. Diabetes mellitus is an independent risk factor for both COVID-19 as well as mucormycosis. At this point, there is scanty data on the subject and most of the published literature comprises of either case reports or case series with no long-term data available. The aim of this review paper is to present the characteristics of COVID-19 related mucormycosis and associated clinical features, outcome, diagnostic and management strategies. A prompt diagnosis and aggressive treatment planning can surely benefit these patients.

## 1. Introduction

There is a deep crisis due to the coronavirus disease (COVID-19) pandemic emerging in the last two years and post COVID-19 infection, such as mucormycosis, is adding further burden to already strained healthcare systems in some countries. Earlier in 2021, in the midst of the pandemic, COVID-19-related mucormycosis had been reported all around the world and 70% of those cases were in India in patients with pre-existing conditions or diabetes (94%). COVID-19 patients with diabetic ketoacidosis, cancer, organ transplant, neutropenia, corticosteroid usage, and hemochromatosis were more likely to acquire mucormycosis [1,2,3].

Mucormycosis or black fungus is a non-septate filamentous fungal infection that causes potentially life-threatening conditions. This typical infection affects immunocompromised and diabetic patients most of the time and the symptoms of this deadly infectious condition depend on the site of origin, but generally facial structures (nose, sinuses, eye, and brain) are most involved. The symptoms associated with rhino-orbital-cerebral mucormycosis (ROCM) are of varying degree (runny nose, unilateral or bilateral facial swelling, orofacial pain, low to high grade fever, headache, blurred vision due to proptosis and involvement of orbital contents, loosening of teeth, destruction of periodontal tissue and appearance of black necrotic eschar or dead bone in the palate, buccal vestibule or the maxillary alveolus along with formation of oro-nasal/oro-antral communication). Although, in the orofacial and maxillofacial region mucormycosis is very rare especially in healthy, immune competent individuals but immune compromised are quite vulnerable to these opportunistic infections which can involve soft and hard tissues of the facial skeleton necessitating surgical intervention and high-dose, long-term parenteral antifungal therapy [4]. A fungi group of molds known as mucormycetes [5,6] cause mucormycosis, which are spread in our environmental air but is more abundant in soil associated with decaying wood, rotten leaves, compost piles, and animal dung [5,6]. The major route of infection spread is via inhalation, which then involves lungs and paranasal sinuses [7]. Treating COVID-19 patients with haphazard medication/self-medication of steroids, antibiotics and zinc may have promoted the dysbiosis of gut microbiota which resulted in inducing immunosuppression and rapid emergence to this mycotic disease [8,9]. In the present scenario, the highest risk to fungal mucoromycetes infection is in those patients diagnosed and treated for COVID-19 with broad-spectrum antibiotics, non-invasive ventilation and received corticosteroid therapies. The patients who had pre-existing diseases, such as asthma, diabetes mellitus and chronic renal failure, and developed COVID-19 on top of it are particularly predisposed to contracting mucormycotic infection.

Although mucormycosis is reported rarely in the localized forms, more recently, several publications have described the clinical management and outcome of mucormycosis infection in the maxillofacial region, for example, the tongue [10], palate [11], mandible [12,13], maxilla [14], and orbitomaxillary/infra-orbital [15,16] region. Therefore, mucormycosis should be considered as a possible diagnosis in case of any spontaneous soft tissue necrotic lesions of orofacial area. In head and neck sites, mucormycosis begins by involving maxillary bone or nose and later directly extends to paranasal sinus and from there, spreads to retro-orbital tissues and can disseminate to eye, brain, lungs and to other body organs [17]. Therefore, it is crucially important to understand the etiology to make an early diagnosis to provide an optimum treatment of the underlying predisposing factors and appropriate medical and surgical interventions [15]. This paper discusses some of the important risk factors, pathophysiology, clinical presentation and outcomes of mucormycosis in patients infected with COVID-19, and several therapeutic regimes used for treating mucormycosis are also presented in this review. The recommended Scale for the Assessment of Narrative Review Articles (SANRA) guidelines [18] were used for reporting this narrative review. Different databases (PubMed and Google Scholar) were searched for the identification of the most relevant literature on COVID-19, mucormycosis and fungal infections.

## 2. COVID-19 and Dentistry

The COVID-19 pandemic has imposed a significant impact on the healthcare system, including dental care practise. COVID-19, caused by the SARS coronavirus 2 (SARS-CoV-2), is presumed to transmit by close contact via respiratory droplets and aerosols. Dentistry is assumed to be involved with the nosocomial transmission of infection due to certain aspects of dental treatment, such as aerosol production and close closeness to patients. The likelihood of bidirectional infection transmission between patients and dental care professionals necessitates additional cautious measures to limit the spread of COVID-19. It is critical to recognize that the rules for delivering dental care during the COVID-19 pandemic will differ around the globe, and dental clinics should follow their area recommendations. This pandemic has also highlighted some of the significant gaps in dental research, as well as the need for new relevant information to handle the current crisis and reduce the impact of future epidemics on dentistry. To summarize, COVID-19 caused several acute issues for dentistry, some of which may have long-term implications for clinical practise, dental education, and dental research [19,20].

In terms of economics, a cross-sectional study was carried out in Nepal and revealed that a large number of dental practitioners (70 percent) were badly impacted by the financial load and were not paid throughout the lockdown. Only 349 (86%) of dentists believed that normal dental treatments should be performed, whereas only 101 (25%) believe that dental emergency treatments for COVID-19 infected patients should be performed [21].

The influence of COVID-19 on urgent dental treatment at the University Hospital Munich and Bavaria, Germany, was investigated in a research study. Patient numbers without and with positive/suspected COVID-19 infection, reasons for attendance and treatments were documented retrospectively and connected to local COVID-19 infection statistics, control measures, and numbers/reasons for private dental office closures. The number of patients in the urgent care unit and private dental clinics fell, followed by a complete recovery. While non-emergency visits were essentially non-existent during the initial lockdown, pain-related therapies were routinely delivered to individuals with positive/suspected COVID-19 infections. The most common reasons for practice closures were a shortage of personal protective equipment (PPE), a lack of personnel, higher health hazards for staff, and infected staff, accounting for 0.72% of all closures (3.6% closures in total). Even in times of high infection risk, pain-driven urgent dental treatment remains a constant requirement, and precautions put in place at the start of the pandemic appear to have created a safe environment for both patients and oral health care professionals. PPE storage is critical to ensuring patients’ treatment in high-risk situations, and its storage and administration by regulatory units may provide a stable and safe oral health care system in the future [22].

A cross-sectional study carried out in Spain found that the return to work of dental hygienists entailed several techniques targeted at limiting infection and ensuring the safety of patients and the rest of the dental team. Personal protective equipment availability, clinical infrastructure adaptability, and patient care management have differed across experts working in the commercial and public sectors [23].

## 3. COVID-19 Related Fungal Infections (Pulmonary and ROCM)

During the COVID-19 pandemic, the illness has been causing yet another major health catastrophe in India. As of May 2021, the Indian government stated that about 12,000 individuals were undergoing treatment for mucormycosis. Many Indian media sites have dubbed it “black fungus” due to the fungus’s ability to create black staining of dead and dying tissue. Even before the COVID-19 epidemic, mucormycosis in India was believed to be 70 times greater than the other part of the globe [24,25].

During the COVID-19 pandemic in 2020 and 2021, a number of instances of mucormycosis, aspergillosis, and candidiasis were connected to immunosuppressive therapy [26]. In early 2021, one review pertaining to the connection between mucormycosis with COVID-19 identified eight instances of mucormycosis, three from the United States, two from India, and one each from Brazil, Italy, and the United Kingdom [27]. The BBC reported an upsurge in instances in India in May 2021 [28]. Diabetes was the most frequent underlying medical condition [27]. Most patients who were hospitalized with severe respiratory issues caused by COVID-19 had recovered but then acquired mucormycosis within 10 to 14 days. Among these patients, five had abnormal kidney function tests, three had sinus, eye, and brain involvement, three had lung issues, one had gastrointestinal (GI) tract involvement, and one had extensive illness [27]. Mucormycosis was diagnosed during the post-mortem in two of the seven fatalities. Because none of the three had conventional risk indicators, the authors questioned the use of steroids and immunosuppressive medications [27]. In a study of COVID-19-related ocular problems, ophthalmic mucormycosis was discovered to occur up to several weeks following recovery from COVID-19 [26].

## 4. Mucormycosis

Baker [29], an American pathologist, created the term mucormycosis in 1957 for a severe Rhizopus infection. Mucormycosis is a rare but deadly fungal illness that often affects people with compromised immune systems. Mucormycosis is an angioinvasive illness caused by mold fungus of the genera *Rhizopus*, *Mucor*, *Rhizomucor*, *Cunninghamella*, and *Absidia* of the Mucorales Order, Class *Zygomycetes* [30]. The most prevalent variety, *Rhizopus oryzae* (*R. oryzae*), is responsible for almost 60% of mucormycosis infections in humans, as well as 90% of the ROCM variant [31]. The inhalation of fungus spores is the mode of contamination.

Mucormycosis is divided into five major kinds based on the area of the body afflicted [32]. Kidney mucormycosis [33] or mucormycosis affecting at other locations but is less frequent, has been characterized as a sixth kind [32].

▪ ROCM; prevalent in patients with uncontrolled diabetes or after a kidney transplant [34,35].
▪ Pulmonary; prevalent in cancer patients or those underwent stem cell or organ transplant.
▪ GI; prevalent in premature as well as low-birth-weight infants receiving medicines, surgery, or drugs that reduce the body’s immune response [36,37].
▪ Burn or other skin damage in patients with leukemia, poorly managed diabetes, graft-versus-host disease, human immunodeficiency virus (HIV), or intravenous (IV) drug abuse [38].
▪ Widespread (disseminated), spreads to other body parts through the bloodstream.

### 4.1. Etiopathogenesis/Pathogenesis

**Figure 1:**
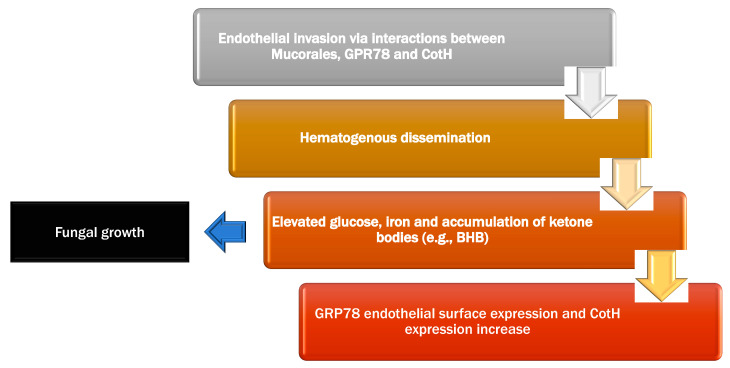
Pathogenesis of mucormycosis. β-hydroxybutyrate (BHB); Glucose regulator protein (GPR78); Spore-coating protein family (CotH).

Mucorales may enter the body via contaminated food ingestion, inhalation, or abraded skin regions. It can induce infections in the ROCM, pulmonary, GI, or cutaneous/wound systems. Mucorales have various characteristics (innate thermotolerance, ability to attach endothelial cell membrane, rapid growth, ability to obtain iron from the host organism, downregulation of host–defense genes associated to pathogen recognition, immune response, and tissue healing), all of which contribute to the disease’s aggressive nature [39,40]. Inhibition of interferon expression [41], as well as an evolutionary duplication of a mechanism involved in energy consumption and pathogenicity, were discovered in a whole-genome string of *R. oryzae* [42]. Mucormycosis predisposing factors ketoacidosis and deferoxamine highlight the role of hyperglycemia, iron, and acidifying ketone bodies in Mucorales pathogenicity (Figure 1).

The Mucorales’ virulence factors human pathogens cause illness in the host in two ways: first, infectious bacteria can elude the body’s defense mechanism and survive inside the host, and second, the immunity is perturbed, impairing the host cells. Pathogen virulence factors play an important part in the damage process [43]. Spores inoculate into the host tissue (depending on the entry site such as alveoli or skin), the evasion of macrophage phagocytosis occurs and they germinating to hyphae (the fungus’s angioinvasive form) thereby increasing their load and attaching to the endothelium via specific unique receptors (spore-coating protein family (CotH)) on the Rhizopus species surface and endothelium glucose regulator protein (GRP78) [44]. As a result, Rhizopus has an enhanced capacity to infiltrate host tissues, explaining the vulnerability of diabetic and deferoxamine-treated individuals to mucormycosis. It should be emphasized, however, that the majority of research on virulence and the relationship between ketoacidosis and the incidence of mucormycosis has been carried out with Rhizopus [45]. Another aspect that contributes to the poor prognosis is Mucorales’ natural resistance to existing antifungals agents (amphotericin B, Posaconazole, itraconazole, and isavuconazole) [46]. Moreover, fungal spores are easily dispersed by aerosolization, local inoculation (e.g., skin lesion), or GI intake. Regardless of the source of entrance, the fungus must be established and mucormycosis must develop. Mucorales spore germination is known to be inhibited by a decrease in the quantity and function of monocytes and neutrophils. Patients with hematological diseases, HIV, or liver cirrhosis, as well as those who have had solid organ transplants and are being treated with high-dose steroids, fall into this category [46,47].

#### 4.1.1. Endothelial Interaction

Mucorales bind to endothelial cells through the expression of CotH proteins [48]. Endothelial cells form the interior layer of blood vessels and play variety of critical roles in pathogen detection and maintaining physiological functions [49], including the ability to phagocytose and destroy Mucorales spores. The receptor GRP78 is found on the endothelial cells surface that may detect Mucor species. In the in-vivo research, increasing the glucose and iron content in mice resulted in increased GRP78 expression on the endothelial cells surface in multiple organs (sinus, brain, and lungs) than the control [50]. CotH proteins are present exclusively in Mucorales and attach to the host endothelial receptor GRP78, resulting in fungus endocytosis after endothelial cells are exposed to acidosis and increased iron and glucose levels (hyperglycemia and diabetic ketoacidosis), both GRP78 endothelial surface expression and CotH fungal surface expression increase [51].

Several clinically relevant observations on the interplay of these receptors are reported in the literature. Acidosis caused by β-hydroxybutyrate (BHB) (a ketone body representative) and higher blood iron levels were the primary variables that increased expression of GRP78 and CotH, but lactic acidosis had no effect on their expression. Furthermore, sodium bicarbonate changed the effect and protected BHB–treated mice from mucormycosis, suggesting the importance of managing acidosis as a therapeutic strategy in diabetic ketoacidosis and mucormycosis [52]. Another study found that either anti-GRP78 or anti-CotH antibodies entirely prevented *R. oryzae* endothelium invasion [53]. This activity, however, suggests the presence of other components implicated in the interaction between endothelial cells and fungus [53]. The development of secondary fungal compounds that function as toxins is another potential cause of endothelium injury [44].

#### 4.1.2. Uptake of Iron

The fact that fungal cells experience apoptosis in low iron circumstances lends credence to the importance of iron in fungal cell growth [54]. Furthermore, a mouse model of mucormycosis, an increased iron concentration promoted fungal growth by decreasing phagocytosis and IFN–production [55]. Mucorales obtain iron from the host via two probable methods: high-affinity iron permeases or siderophores [56]. The presence of heme-oxygenase copies in *R. oryzae* or genome sequencing point to a third method of iron absorption from hemoglobin seen in fungus [42].

Deferoxamine, an iron chelator used in people at high risk of iron overload (e.g., patients on renal replacement therapy and those receiving repeated transfusions), makes people more susceptible to mucormycosis [57]. Subsequent research has suggested that iron chelation therapy with deferasirox or deferiprone protects diabetic ketoacidosis mice from mucormycosis and improves survival, while an adjunctive deferasoxinanophen label study of eight mucormycosis cases showed beneficial results [57]. Nevertheless, a clinical study in individuals with mucormycosis reported that used supplementary deferasirox treatment failed to demonstrate a survival advantage [58]. Mucorales have ferrioxamine receptors (Fob1 and Fob2) that are activated only in the ferrioxamine presence, allowing fungal iron absorption. Iron absorption from ferrioxamine is energy-dependent due to the action of reductase, which liberates ferric iron from deferoxamine extracellularly and converts it to soluble ferrous iron, as well as full intracellular ferrioxamine uptake [58].

FTR1, a high-affinity iron permease, has been proposed to promote intracellular iron transport from heme or ferrioxamine. It is expressed in iron-depleted settings but inhibited in iron-rich situations [48]. Acidic sera that promoted *R. oryzae* growth had more accessible serum iron (69 g/dL versus 13 g/dL for sera not supporting *R. oryzae* growth). Lastly, induced acidotic circumstances reduced the iron-binding capacity of sera obtained from healthy individuals, suggesting that acidosis momentarily impairs transferrin’s ability to bind iron [59].

#### 4.1.3. Interaction between Mucorales and Immune Defense

Evidence on the relationship between the most common organisms resulting in mucormycosis and the role of immune cells is summarized as below:

##### Platelets

Platelets have an essential function in host immunity, which is well established in the literature [59]. Following the fungi exposure, granules containing pro-inflammatory cytokines and chemokines, including thrombocidins and transforming growth factor-B with fungicidal characteristics, are secreted [60]. Membrane-bound molecules (CD154 and platelet Toll-like receptors) are expressed, allowing platelet binding and activation of different cells and their functions:

Endothelial cells stimulate the intracellular adhesion molecule-1 and vascular cell adhesion molecule-1 pathways.

Monocytes can be activated or differentiated into macrophages.

Dendritic cells stimulate their maturation, whereas B and T lymphocytes stimulate their activation.

Mucorales spores and hyphae promote platelet activation and enhanced aggregation, clot formation, and platelet consumption, which causes fungal harm by inhibiting hyphal development [61]. Besides, platelet aggregation to the fungal wall might inhibit fungi from spreading hematogenously. Furthermore, necrotic regions in organs that do not have fungal development imply thrombotic ischemia, which might be caused by systematic platelet activation.

##### Natural Killer (NK) Cells

NK cells are lymphocytic and have a variety of receptors that may detect diseased cells and block major histocompatibility complex (MHC) that inhibits receptor activation [62]. NK cells are a kind of innate immunity having both direct and indirect cytotoxic abilities. Chemokines and cytokines (IFN-, TNF-, and GMCSF) are also secreted by these cells [63]. However, in-vitro investigations have revealed that *R. oryzae* has an immunosuppressive impact, preventing the release of immune regulatory chemokines from NK cells [64].

##### T Cells

Antigen-specific T lymphocytes are type of adaptive immunity and a promising diagnostic tool for infectious disease control [65]. Mucorales-specific T cells have been shown to be detected in the majority of mucormycosis patients as compared to other individuals who generated the cytokines IFN-, IL-4, IL-10, and IL-17 these cytokines caused Mucorales hyphal destruction [65].

T-inactivated cells treated with cytokines IL-2, IL-7, or both, produce more Mucorales-specific T cells and their cytokines IL-5, IL-10, and IL-13, as well as CD4+ T cells that recognize particular Mucorales antigens [66].

**Figure 2:**
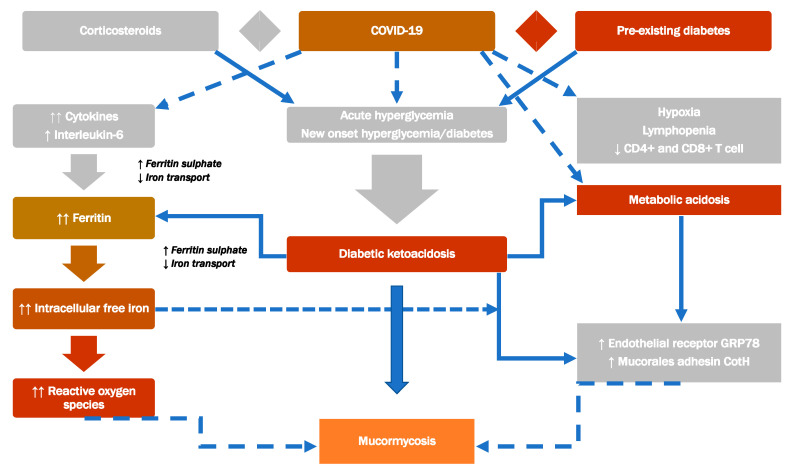
Association of diabetes, corticosteroid, and COVID-19 with mucormycosis. Reprinted with permission from ref. [67]. Copyright Year (2021) Copyright Owner’s Name (Elsevier). Glucose regulator protein (GPR78); Spore-coating protein family (CotH).

Figure 1 shows the pathogenesis of mucormycosis, and Figure 2 demonstrates the proposed interaction of diabetes, corticosteroid, and COVID-19 with mucormycosis.

### 4.2. The Most Common Risk Factors for Mucormycosis

Mucormycosis is often an opportunistic infection with particular risk factors; however, a minor proportion of infections also occur in healthy individuals [1,68]. People at greatest risk of developing invasive disorders have reduced numbers of mononuclear and polymorphonuclear phagocytes, as seen in neutropenia, or disorders that affect the phagocyte function, as in hyperglycemia/acidosis or glucocorticoid administration. These factors weaken the immune system and allow fungus to grow and spread, resulting in invasive illness (Figure 2). Table 1 lists the important conditions predisposing to mucormycosis development. Moreover, Table 2 shows the factors of immunocompetent individuals developing mucormycosis.

### 4.3. Mucormycosis Clinical Manifestations

The clinical features of mucormycosis vary depending on where the infection is located. Generally, infection starts in the oral cavity or nose and travels to the central nervous system through the eyes [38]. If the infection spreads from the sinuses or nose to the brain, clinical features may involve unilateral eye discomfort or headache, as well as facial pain, numbness, fever, anosmia, and a runny or blocked nose. The symptoms may resemble sinusitis individual. One facial side may seem enlarged, with quickly developing “black lesions” through the palate or nose. One of the eyes might appear enlarged and bulging, with blurring of vision, diplopia or decreased visual acuity [69]. When the lungs are affected, symptoms such as pyrexia, chest discomfort, cough, dyspnea, and hemoptysis might develop. When the GI tract is affected, symptoms such as stomach pain, nausea, vomiting, and bleeding may ensue. Due to tissue loss, the skin affected may look like a darkish red sensitive area with a deepening center. There might be an ulcer, which can be quite uncomfortable [38,68,70].

Invasion into blood arteries can cause thrombosis and eventual death of nearby tissue owing to a lack of vascular supply [68]. Because disseminated mucormycosis is often present in patients with pre-existing medical problems, it may be challenging to determine which symptoms are linked to mucormycosis. Patients who have a disseminated mucormycosis in the brain may experience changes in the mental status or go into a coma [39,68]. One of the initial presentations of ROCM may be multiple mobile teeth with gingival erythema and pus discharging sinuses. These manifestations mimic odontogenic infection and has been a source of missed diagnosis by the general dentists who are not familiar with the clinical presentation of this deadly disease. It has been observed that general dental practitioners, who had low index of suspicion for mucormycosis, have wasted precious time in attempting root canal therapies and performing tooth extractions of these mobile teeth leading to delay the commencement of definitive treatment of mucormycosis and thus resulting in a poor/fatal outcome for the patient [71,72].

A review by Hussain et al., [73] described the clinical presentation, treatment methods, and patient outcomes of complementary and alternative medicine for COVID-19 associated mucormycosis. According to this review, diabetes mellitus (73.65%), hypertension (22.75%), and kidney failure (10.77%) were the most frequent co-morbidities among COVID-19 associated mucormycosis patients. Moreover, facial discomfort, ptosis, proptosis, visual acuity, and vision loss were the most prevalent complaints identified. Patients who received both medicinal and surgical care had a better chance of survival (64.96%). The overall death rate among these patients was found to be 38.32%. To decrease morbidity and mortality associated with COVID-19 associated mucormycosis, optimal glycemic management and early detection of mucormycosis should be prioritized [73].

### 4.4. Mucormycosis Diagnosis

Diagnosis necessitates detecting the mold in the afflicted tissue through biopsy and a confirmatory fungal culture [56,74]. As the causal fungi are found everywhere, cultivation alone is not sufficient [38]. Culture as well as direct detection of the pathogen in body fluids such as blood, serum, plasma, lung fluid, and urine are additional possible tests [75]. Complete blood levels are performed as part of the blood tests to look for neutropenia. Levels of blood glucose, iron, bicarbonates, and electrolytes are among the other blood tests. It is possible that an endoscopic evaluation of the nasal passages will be required [3,76].

#### 4.4.1. Clinical Diagnosis

An identification of host variables, quick evaluation of clinical symptoms, and a strong index of suspicion are required for the diagnosis of mucormycosis. Pleuritic discomfort in a neutropenic patient or diplopia in a diabetic patient are the symptoms of mucormycosis infection that prompt the use of diagnostic imaging techniques and the following collection of samples for testing by microbiology, histology, and sophisticated molecular modalities. Mucormycosis is distinguished by tissue necrosis; nevertheless, the presentation and syndrome-oriented method to diagnosis is insufficient in terms of specificity and sensitivity. Other funguses, for example, *Fusarium* or *Aspergillus*, can cause similar clinical symptoms. Furthermore, in tuberculosis-endemic nations, the two diseases can coexist, as seen in a diabetic patient [77]. Nonetheless, there are several characteristics that should raise the bar for invasive mucormycosis of lungs. These comprise a history of previous voriconazole prophylaxis or the appearance of a breakthrough fungal infection in an immunocompromised individual receiving *Aspergillus* but not Mucorales-specific medications [78]. Corzo-Leon et al. devised a method for detecting ROCM in diabetic individuals. The following clinical manifestations must be regarded “red flags” which includes diplopia, proptosis, sinus discomfort, periorbital edema, cranial nerve palsy, palatal ulcers, and orbital apex syndrome [79].

Since most of the presenting signs and symptoms are not specific for fungal sinusitis, it is recommended that clinicians and dental practitioners should be cautious and vigilant [80], and should maintain a low threshold for referral to an oral and maxillofacial surgeon or otorhinolaryngologist especially if a patient has a history of COVID-19 infection in the past where he/she was hospitalized and was administered high-dose systemic steroids, broad anti-microbials and mechanical ventilation presents with any of these red-flags signs and symptoms. This can save the life and vital organs, such as eyes, in a patient who has post-COVID mucormycosis [81].

#### 4.4.2. Microscopic Examination and Culture

The pillars of diagnosing mucormycosis are direct and histopathological microscopy, and cultures of different clinical samples. Direct microscopy of clinical samples, particularly with optical brighteners, such as Blankophor [82] and Calcofluor [83] White, provides for a quick and plausible mucormycosis diagnosis [84]. Mucorales hyphae are non-septate or pauci-septate, vary in size and have an uneven, ribbon-like manifestation. Fungal elements are clearly visible on periodic acid-Schiff; hematoxylin and eosin sections or Grocott-methenamine. Gomori’s silver is used to highlight fungal hyphae and thus analyze greater detail of morphology [83]. Inflammation, whether neutrophilic or granulomatous, dominates tissue histology; nevertheless, inflammation might not be present in some cases, notably in immunocompromised people [68]. Invasive lesions are distinguished by angioinvasion and large infarcts. A perineural invasion may be evident when nerve structures are implicated. In comparison to non-neutropenic individuals, neutropenic individuals have more widespread angioinvasion [82]. Histopathological analysis may not always provide a clear distinction between *Aspergillus* or morphologically similar fungus hyphae and Mucorales hyphae. Tissue identification, on the other hand, is a critical diagnostic technique as it separates the fungal existence in the material from a culture contaminant. On the majority of fungal culture medium, such as Sabouraud agar and potato dextrose agar cultured at 25 °C to 30 °C, all Mucorales grow quickly (3 to 7 days) [85,86]. A microaerophilic environment enhances culture yield for some species [87]. Despite the presence of fungal hyphae in histopathologic examination, cultures are only positive in half of the cases [3]. Because hyphae are of friable nature, they can be destroyed during manipulation of tissue. Therefore, evasion of excessive tissue homogenization is suggested.

For immunohistochemistry examination, a particular mouse monoclonal anti-Rhizomucor-antibody has been used; nonetheless, this test has formerly been demonstrated to react with other Mucorales and Entomophthorales [88]. In situ hybridization targeting 5S and 18S ribosomal RNA sequences [89] is still under research.

#### 4.4.3. Antifungal Susceptibility Testing and Identification of Species

Identification of species is important for a comprehensive epidemiological understanding of mucormycosis and can be useful in epidemic investigations. Mucorales may be easily distinguished from *Aspergillus* in cultivation. A study revealed that when examined by persons with experience of fungal identification, morphological characteristics alone may provide a higher degree of accuracy [90]. Nevertheless, this is challenging and may be linked to speciation failures [91]. ID32C kit (bio Merieux, Marcy lÉtoile, France) has been successfully used to identify *Lichtheimia corymbifera* and *R. pusillus*, and API 50CH (bioMerieux, Marcy-l’Étoile, France) [92] for Mucor species. Both tests failed to differentiate *M. circinelloides* and *M. rouxii. L. ramosa* is detected using ID32C and positive melezitose assimilation [93]. Although matrix-assisted laser desorption/ionization time-of-flight mass spectrometry (MALDI-TOF) is a favorable technique, it has not yet been verified for every Mucorale [94]. Additionally, a dependable technique is to use molecular-based tests that focus on the internal transcribed spacer region [91].

*M. circinelloides* has a greater minimum inhibitory concentration (MIC) in contradiction of posaconazole, whereas *Cunninghamella* and *Rhizopus* have a greater MIC against amphotericin B [95]. Few *Apophysomyces* isolates have a greater MIC for amphotericin B [90]. The relevance of this information in patient care is unknown, but it needs to be investigated further.

#### 4.4.4. Serology

Enzyme-linked immunosorbent assays [96], immunoblots [97], and immunodiffusion tests [98] are being tried with varying degrees of success. An enzyme-linked immunospot (ELISpot) test identified Mucorales specific T lymphocytes in three hematological patients with invasive mucormycosis [66]. Mucorales-specific T lymphocytes were not seen in any of the controls. Further research will be conducted on the use of such particular T cells as surrogate diagnostic indicators.

#### 4.4.5. Molecular Assays

Standard polymerase chain reaction (PCR) [99,100], restriction fragment length polymorphism analyses (RFLP) [101,102], DNA sequencing of specified genes [103,104], and melt curve analysis of PCR products are examples of molecular-based tests [105]. All of the assays mentioned above can be used to detect or identify Mucorales. The bulk of molecular tests focus on the internal transcribed spacer or the 18S rRNA genes [88,90]. Various investigations have been conducted utilizing either formalin-fixed, paraffin-embedded, or fresh tissue samples [88], with varying results. Sensitivity (70–100%) and specificity (not estimated to 100%) varied among investigations, with the main drawback being the small number of individuals investigated. The efficacy of these in-house tests has not been extensively researched, and there has been insufficient clinical assessment; therefore, they cannot be advocated as an individual, single method in routine clinical investigations [88]. Lately, efforts at molecular diagnostics from blood and serum 58–60 have generated encouraging clinical results. When compared to culture, molecular-based diagnosis from serum led to earlier diagnosis and overall verified culture-proven instances. Molecular-based diagnostic tests are currently suggested as important add-on tools that supplement traditional diagnostic methods [88,91,105].

#### 4.4.6. Imaging

Imaging, such as CT scans of the sinuses and lungs are frequently performed [106]. Signs on chest CT scans, comprising cavities, nodules, pleural effusion, halo signs, and wedge-shaped shadows, displaying blood vessels invasion may imply a fungal infection, though not confirming mucormycosis [107]. A reverse halo sign (RHS) in a patient with a reduced neutrophil level and blood cancer is strongly suggestive of mucormycosis [107]. CT scan pictures of mucormycosis may be used to differentiate between orbital mucormycosis and orbital cellulitis, although the imaging may appear similar to Aspergillosis [107]. MRI may also be beneficial [26]. MRI with gadolinium contrast is now the method of choice in ROCM.

In recent research that used consecutive thoracic CT scans on leukemic patients with low neutrophil count, the RHS was seen in 94% of patients during the first seven days of the illness, but other radiographical abnormalities, like numerous nodules, emerged latter. The RHS presence on CT was a significant indication of pulmonary mucormycosis in the specific context of neutropenic leukemic individuals with lung infection [108]. Another research compared CT scans from 24 individuals with pulmonary mucormycosis to those from 96 patients with invasive Aspergillosis of lungs. The RHS was more prevalent in mucormycosis patients (54%) than in Aspergillosis patients (6%), although several airway-invasive characteristics, such as clusters of centrilobular nodules, peribronchial consolidations, and bronchial wall thickening, were more common in aspergillosis patients [109]. While these findings are not definitive, they might be utilized as a trigger to initiate aggressive diagnostic laboratory investigations. Positron emission tomography-computed tomography (PET/CT) with [18F]-fluorodeoxyglucose (FDG) is another developing imaging technology that may ultimately help in the diagnosis and management of mucormycosis [110]. Endobronchial ultrasound-guided fine needle aspiration is also a valuable diagnostic technique when it is possible [111].

In ROCM, as mentioned earlier, contrast enhanced MRI is the imaging of choice. Presentation of classic ‘black turbinate’ sign on axial or coronal slices shows fungal rhino-sinusitis. Non-enhancing lesions of the sinus and extra-sinusal tissues are also seen. Angio-invasion and fungal vasculitis present as infarction of internal carotid artery, central artery of retina, and cerebral arteries. Contrast enhanced scans can show areas of devitalized tissues in and around the orbits, maxillary, and the ethmoidal sinuses. Similarly cavernous sinus thrombosis can present as non-enhancing lesion on a contrast-enhanced fat-saturated MR image. Intra-cranial extension can present as a hypointense dural enhancing lesion. The usual signs, which suggest fungal involvement on a CT scan, include partial or complete opacifications/haziness of one or more of the para-nasal sinuses, appearance of a separation line between healthy and necrotic bone, which can present with mobility of the teeth, sequestrum formation in the maxilla or zygomatic bone, involvement of the orbital contents, soft tissue prominence and effacement of fat in and around pterygopalatine fossa [112,113].

### 4.5. Clinical Cases of Mucormycosis

**Figure 3:**
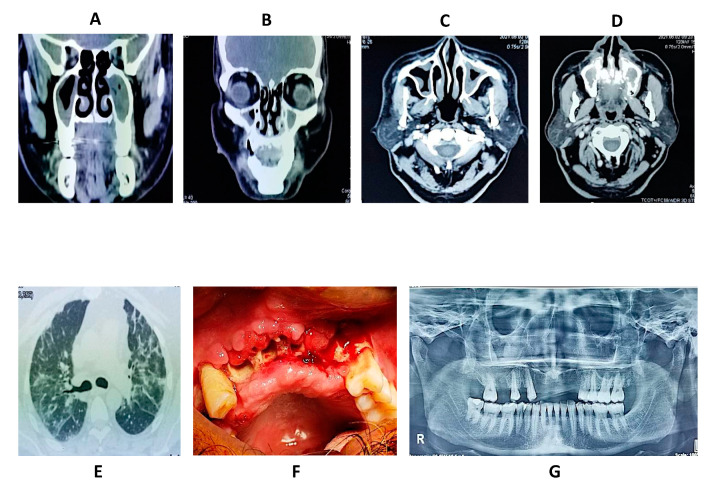
Coronal slices of CT scan with sinus opacification and sequestrum formation (**A**) and (**B**). Axial slices of the contrast enhanced CT scan and show involvement of maxillary sinuses and nasal turbinates (**C**) and (**D**). An axial slice of HRCT of chest showing post-COVID fibrosis (**E**). A clinical picture showing non-healing extraction sockets and necrotic bone in the maxilla, the rest of the teeth present were grade I mobile (**F**). OPG of the same patient showing sinus involvement, thickening of the lamina dura of the extraction sockets in the anterior maxilla (**G**).

**Figure 4:**
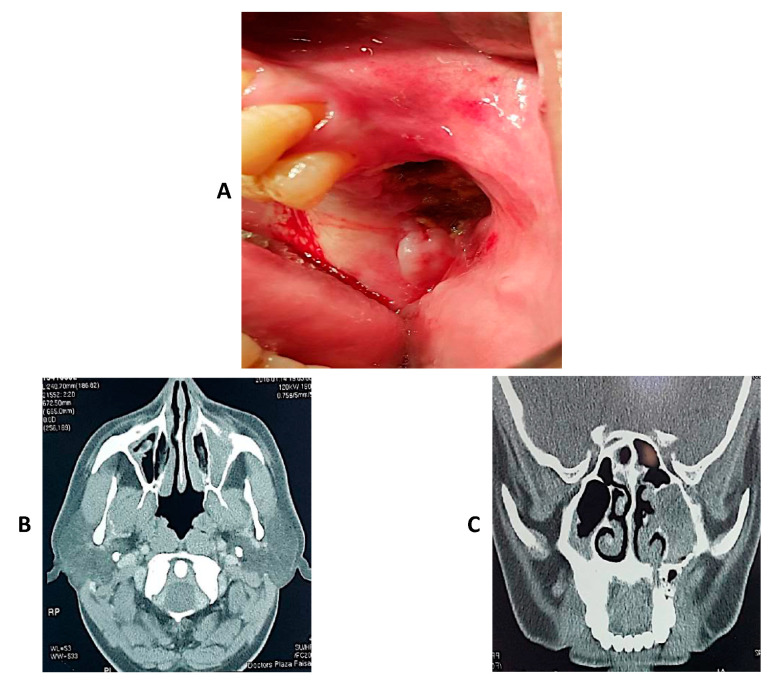
Picture A, showing exposed necrotic bone in the left posterior maxilla (**A**). The axial (**B**) and coronal (**C**) slices of contrast enhanced CT scan showing bone destruction, sequestrum formation, sinus opacification, and involvement of the nasal turbinates.

**Figure 5:**
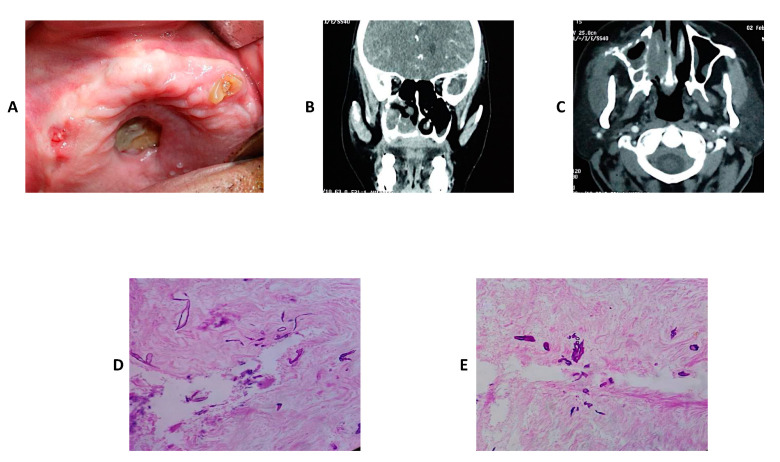
An edentulous maxilla with an exposed necrotic bone in the palate and draining sinus in the right canine region (**A**). The axial (**B**) and coronal (**C**) slices of contrast enhanced CT scans of the maxilla respectively showing bone destruction, sequestrum formation, involvement of the right turbinate and sinus opacification. Histopathological slides (H&E staining) showing non-septate fungal hyphae consistent with diagnosis of mucormycosis (**D**,**E**).

In this section, three cases of mucormycosis are shown in Figure 3, Figure 4 and Figure 5.

### 4.6. Oral and Dental Manifestations

Table 3 demonstrates the oral and dental manifestations of ROCM [114]. Ahmed et al. described a case series of 21 post-COVID-19 people (2 weeks after recovery) with oral mucormycosis (11 males (52.4%) and 10 females (47.6%)) with a mean age of (58 ± 12) years. They observed that in COVID-19 individuals, oral signs of mucormycosis are often evident in the palate and may include varied degrees of mucosal staining, swelling, ulcerations, superficial necrotic regions in the palate, bone exposure, and necrosis with black eschar development. As a result, palatal ulcerations may be the first presenting symptom, prompting the patient to seek treatment from a dentist, who may be the first clinician to identify an infection, leading to the diagnosis of mucormycosis [115].

## 5. Management of COVID-19 Related Fungal Infections

### 5.1. Prevention of Mucormycosis

It may not be possible to prevent development of deep invasive fungal infections in predisposed patients, such as uncontrolled diabetics, transplant patients, patients with chronic sinusitis or previous history of mucormycosis, HIV patients or those taking steroids however incidence of mucormycosis in COVID-19 patients may be reduced if certain precautions are undertaken. These may include [116]:

▪ Promoting personal hygiene, including general hygiene like that of hands and face as well as the oral hygiene through proper brushing and antiseptic mouth rinses (chlorhexidine 0.2% or betadine)
▪ Delivery of oxygen should be in a strictly aseptic environment with regular changing of filters and tubes as they can harbor fungus, if contaminated.
▪ Aggressive management and monitoring of immunocompromised state like in case of diabetes, monitoring of blood sugar levels and employing strict control.
▪ Using steroids very carefully, under strict control and according to recommended guidelines.
▪ Caution in using tocilizumab and other related agents.
▪ Consider prophylactic oral delayed release posaconazole (600 mg day 1, 400 mg 2 to 14 days and 300 mg for 3-months) or isavuconazole (200 mg q8H for 1- to 2-days and 200 mg/day for 3-months) [117].

### 5.2. Treatment of Mucormycosis

Management of mucormycosis depends on early diagnosis with a clinician possessing a high index of suspicion, optimization of the underlying disease, thorough debridement, and supportive anti-fungal medication. A mainstay of treatment of invasive fungal infections remains surgical debridement supported with anti-fungal drugs, with reversal of immune compromised state affecting the final outcome. Medical management comprise of broad spectrum anti-fungal agents and systemic anti-microbials for prevention against superadded bacterial infections [116,118]. Details of medical and surgical treatment are provided below.

#### 5.2.1. Medical Treatment

Several types of antifungals have demonstrated efficacy in the treatment of mucormycosis (Table 4).

##### Systemic Agents

**A. Amphotericin B:**

Amphotericin B is a polyene antifungal agent. It comes as either amphotericin B deoxycholate, also referred to as conventional amphotericin B and newer lipid formulations, which are considered less toxic than the conventional variety [119]. These newer formulations include liposomal amphotericin B, amphotericin B lipid complex, and amphotericin B cholesteryl sulphate complex. The benefit of these lipid formulations is lesser toxicity, better drug delivery to the affected sites and allows provision of a higher daily dose [120]. For many years, Amphotericin B was considered as first line treatment for invasive candidiasis, mucormycosis and aspergillosis however its popularity is somewhat declining with the advent of new azole derived antifungals because of a broader spectrum of activity and a better safety profile. In addition to treatment of mucormycosis and aspergillosis, amphotericin B is also considered a drug of choice for cerebral cryptococcosis, coccidioidomycosis, Para coccidioidomycosis, disseminated histoplasmosis, severe blastomycosis, and visceral leishmaniasis [121].

*Mechanism of Action:*

The principal sterol in fungal cell membranes is the ergosterol. Amphotericin B binds to the ergosterol and opens up ion channels leading to increased cell membrane permeability which in turn produces depolarization of the cell, metabolic disturbances, and leakage of small molecules. The end result of this cascade is destruction of fungal cells. Another mechanism of action is the production of oxidative damage to the cells through creation of free radicals leading to cell membrane damage. Moreover, amphotericin B is thought to enhance the phagocytic properties of macrophages, which improves the clearance of fungus from the body [122].

*Dosing and Administration:*

Fungicidal activity is dose dependent and usually lasts for up to 12 h and the normal dose ranges from 0.7–1 mg/kg/day, which is given slowly over 2 to 4 h as rapid infusion can result in cardiotoxicity. Risk of nephrotoxicity is enhanced if the doses exceed 1 mg/kg. The patients who present with a risk of nephrotoxicity should be administered 1000 mL of normal saline prior to infusion of amphotericin B. Higher doses can be administered (3 mg/kg/day) in case of liposomal amphotericin B because produces less nephrotoxicity and remains bound to liposomes in circulation. Higher than usual doses (10 mg/kg/day) are associated with severe nephrotoxicity and infusion related events hence should be avoided [123,124].

Water-solubility of the drug is virtually absent hence it cannot be given per oral or through intramuscular (IM) injection. Amphotericin B is a skin irritant hence its topical use is not recommended. Amphotericin B produces high concentrations in tissues like liver, spleen, bone marrow, kidney, and lungs. When administered intravenously, the cerebrospinal fluid (CSF) concentration is around 5% of that in serum hence for the treatment of fungal infections of the central nervous system, it is recommended that the drug should be given intrathecally [120,121].

*Spectrum of Activity:*

Liposomal amphotericin B is active against Candida species producing invasive candidiasis or candidemia, aspergillus and zygomycetes and it is approved for use in many countries for the management of invasive fungal infections. For many decades, Amphotericin B deoxycholate was considered the gold standard of treatment for invasive fungal infections. Later these lipid-associated formulations were introduced which have shown a similar degree of efficacy with a better safety profile. Similarly, liposomal amphotericin B is more effective in HIV-related disseminated histoplasmosis and cryptococcal meningitis [125,126].

*Adverse Effects and Contraindications:*

The majority of the toxicity and adverse effects are produced due to interaction of amphotericin B with cholesterol in human plasma membranes. Common adverse effects include hypokalemia (which can be prevented with concomitant use of steroids), hypomagnesemia, severe anaphylactic reactions, nephrotoxicity leading to renal failure, and the need for renal dialysis(which is reversible in most cases), anemia (normochromic/normocytic) and neurotoxicity causing demyelinating encephalopathy. Anaphylactic reaction is an absolute contraindication for administrating amphotericin B. Administration of acetaminophen, diphenhydramine, and hydrocortisone prior to administering amphotericin B can reduce infusion related side effects [127].

*Efficacy in mucormycosis:*

The outcome of the disease is mainly dependent on the dissemination of infection, cerebral involvement, and ability to debride surgically as surgical debridement remains the mainstay of treatment with medications playing only a supporting role. Lanternier et al. treated 40 cases of mucormycosis with surgical debridement along with liposomal Amphotericin B and demonstrated an overall response rate of 36% at 4 weeks of treatment with a slight improvement in response to around 45% at the third month of therapy. They reported 53% mortality at the 6-month follow-up. Another finding highlighted was that in around 63% cases who were treated with liposomal Amphotericin B, these patients presented with an almost two-fold increase in creatinine levels [128].

Rodriguez-Morales et al. recommend that whenever there is suspicion of mucormycosis in COVID related patients, the first line of therapy should be high dose liposomal amphotericin B [129]. Later on, the patients can be switched towards isavuconazole or posaconazole for maintenance therapy. According to their recommendation, conventional amphotericin B should be avoided, if possible, because of high incidence of toxicity however in resource limited settings conventional amphotericin B may be the only agent available. Despite aggressive treatment they have reported a mortality rate up to 70%, depending on the dissemination of disease and organ involvement [129].

It is a general recommendation that kidney function must be monitored when prescribing amphotericin B and second line therapies may have to be considered keeping in view the patient’s response to treatment and organ involvement. Alekseyev et al. suggest combining amphotericin B with echinocandins, such as caspofungin, or with triazoles like posaconazole/isavuconazole as alternatives in cases who are allergic or intolerant to amphotericin B [130].

According to Spellberg, post-infection survival was almost 83% if the anti-fungal treatment with amphotericin B was initiated within 5-days of diagnosis and it drops to almost 49% if the treatment is delayed to more than 6-days hence highlighting the significance of early diagnosis and early initiation of therapy for these patients [68]. Spellberg and his colleagues also reported that liposomal amphotericin B is almost twice as potent and effective as conventional amphotericin B (survival rates 67% and 39%, respectively) [68].

**B. Itraconazole:**

Itraconazole belongs to the group triazole and depicts broad spectrum anti-fungal activity. Once with in the body, itraconazole is converted to its metabolite hydroxy-itraconazole which in itself possesses anti-fungal properties [131]. It is available both in the IV and oral formulations with IV showing better efficacy for deep seated fungal infections [132]. Newer formulations combine a ring of hydroxypropyl-β-cyclodextrin with itraconazole molecule to improve its absorption and bioavailability making them suitable for a wider range of infections [131].

*Mechanism of Action:*

Its mechanism of action is similar to that of fluconazole as it prevents cell membrane function in the fungal cells by inhibiting the synthesis of ergosterol. Precisely speaking, it blocks the conversion of lanosterol to ergosterol by interacting with substrate binding site of fungal cytochrome P-450. Defective ergosterol leads to increased cell membrane permeability and it also adversely affects membrane-bound enzyme activity [131,132].

*Dosing and Administration:*

Itraconazole is available in the form of capsules, oral solution, and IV formulations. The drug is lipophilic hence shows variable absorption when administered in capsule form. However, the absorption from stomach is better if pH of the gastric contents is low, hence it is recommended that it must be taken with food. Its lipophilicity leads to minimal bioavailability in body fluids, such as saliva, CSF, and lacrimal fluid [131]. In body organs, such as skin, lung, liver, and kidney, the drug concentration may increase 20- fold, especially in the skin; therefore, it is most effective in fungal infections of the skin. This property of the drug is utilized in a pulse regimen [133].

*Spectrum of activity:*

As mentioned above, itraconazole is a broad spectrum anti-fungal agent and has been used to treat blastomycosis, aspergillosis, histoplasmosis, paracoccidiodomycosis, coccidioidomycosis and candidiasis. In addition to its therapeutic indication, use of itraconazole has also been recommended by fungal prophylaxis in HIV or other immunocompromised patients, owing to its safety profile and minimal fungal resistance [132,134]. Itraconazole has also been approved by the FDA for treatment of superficial fungal infections, such as onychomycosis, vulvo-vaginal, and oro-pharyngeal candidiasis, and some other topical mycosis.

*Adverse effects and contraindications:*

Reported side effects, including GI disturbances such as gastric discomfort, nausea, vomiting and diarrhea [135]. Other relatively uncommon but somewhat serious adverse effects include resistant hypertension in known hypertensive patients, cardiotoxicity leading to a reduction in contractile forces of the heart and decreased ejection fraction and hepatotoxicity causing raised ALT levels [136]. Therefore, it is recommended that liver enzymes must be monitored if the therapy has to continue for more than 1 month. Its administration can also lead to fever, joint pain, dysgeusia, pruritis, and headache.

Use of itraconazole is contraindicated in heart failure patients because of its potential to cause cardiotoxicity. Similarly, likelihood of causing liver damage makes its use unsafe in patients with chronic liver disease. Azoles are labelled as teratogenic and possess embryotoxic effects, hence the use of itraconazole is contraindicated in pregnant patients because it can lead to ocular defects in newborns [137].

Administration of itraconazole can lead to drug-drug interactions because of its metabolism using CYP 450 pathway in the liver. The common drugs which it can interfere with include terfenadine, astemizole, midazolam, and oral hypoglycemics. 400 mg/day is generally considered as the upper safe limit of itraconazole, and serious side effects are reported at doses of 600 mg/day [135].

*Efficacy in mucormycosis:*

Similar to fluconazole, itraconazole also has a limited efficacy against deep fungal infections caused by mucor species. According to the literature, the efficacy is limited to Absidia species. Data based on animal and human studies regarding efficacy of itraconazole in mucormycosis is limited, hence it is not recommended to use itraconazole as a single agent in the treatment of mucormycosis; however, it can be used as a third line agent in case amphotericin B is contraindicated and posaconazole is unavailable and even in this case, recommendation is to use it as a combination or adjunctive therapy [138]. According to Jeong et al., itraconazole capsules as monotherapy can be prescribed for cutaneous disease in immunocompetent individuals owing to its ability to accumulate in the superficial infection sites on the skin. Some in-vitro studies have shown promising results where itraconazole has shown effectiveness against Mucorales but in most animal studies the results have been somewhat disappointing [139]. Dannaoui et al. studied the efficacy of itraconazole against different species and genera of zygomycetes and found in vitro MIC (90) for itraconazole to be in the range of 0.03 to 32 mg/L. They found that Absidia species demonstrated better response to itraconazole as compared to Rhizopus [140]. An almost similar results for itraconazole were demonstrated by Almyroudis et al. [141]. These drugs are frequently available in a majority of developing countries in Asia and Africa.

**C. Posaconazole**

Posaconazole is a new triazole antifungal agent, which is structurally related to itraconazole and is produced when the chlorine in the phenyl ring of itraconazole is replaced with fluorine. This structural alteration not only enhances its spectrum of activity but also helps in improving its potency. Depending on the dose and on the target organism, posaconazole can be either fungicidal or fungistatic. It is available in IV and oral formulations and is approved for the treatment and prophylaxis of invasive fungal infections and oropharyngeal candidiasis [142,143].

*Mechanism of Action:*

Since posaconazole is structurally an azole, its mechanism of action is similar to the rest of the azoles whereby it exerts its actions principally through blocking enzyme sterol 14α-demethylase in the cytochrome P-450 dependent pathway. This blocking inhibits ergosterol synthesis in the fungal cell membrane thus causing fungal cell lysis. Its fungicidal efficacy has been found to be better than itraconazole in regard to treatment of zygomycetes [144,145].

*Dosing and Administration:*

Posaconazole is available as oral suspension (40 mg/mL), delayed-release tablet (100 mg) and injectable solution (18 mg/mL ~ 300 mg/16.7 ml vial). Oral suspension is usually prescribed as 200 mg three times a day, tablet and IV infusion are administered as 300 mg twice on one day followed by 300 mg OD [146]. These doses are for invasive infections and 100 mg BD on one day followed by 100 mg OD is generally administered for oro-pharyngeal candidiasis. For infections resistant to fluconazole and itraconazole, the dose is usually increased to 400 mg BD. Posaconazole, when administered as a therapeutic regimen, is continued for 3 to 4 weeks, depending on the response and severity of the disease. It is recommended that posaconazole be taken with food, nutritional supplement or with a carbonated beverage as acidic environment enhances its absorption from the stomach [147,148,149].

*Spectrum of activity:*

Posaconazole is highly lipophilic, well absorbed after oral administration, distributed extensively, and is one of the most potent azoles thus far produced. Owing to its structural differences from fluconazole and itraconazole, posaconazole possesses the ability to interact with additional domains of the target making it effective in mutated strains resistant to fluconazole and itraconazole [147]. It has been found to be effective against Candida species, *cryptococcus neoformans, aspergillus* species, fusarium and zygomycetes. As mentioned earlier, posaconazole has been approved for the treatment and prophylaxis of invasive aspergillosis, fusariosis, and zygomycosis, including mucormycosis [150]. At a dose of 200 mg three times a day, posaconazole has been found to be effective against breakthrough fungal infections also. Studies have found statistically significant results in patients treated with posaconazole for invasive fungal infections as compared to itraconazole and fluconazole [145,151].

*Adverse effects and contraindications:*

The most common side effects reported in literature are GI, with diarrhea, nausea, and vomiting being on top of the list. Oral formulations generally lead to lesser side effects than their IV counterpart. There are reports in the literature where IV infusion had to be discontinued because patients developed significant infusion related problems [152]. Headache, thrombocytopenia, fatigue, weight loss, rash, loss of appetite, mucosal inflammation, anemia, edema, nausea, vomiting, and dizziness are other reported side effects related to posaconazole therapy [153].

Clinicians should be cautious when prescribing posaconazole to patients with liver function abnormalities and liver function tests must be monitored during the course of treatment. IV formulations should be avoided in patients with moderate to severe renal impairment (GFR < 50 mL/min). Another thing which must be monitored during the therapy is the serum electrolytes because posaconazole is known to cause hypokalemia and hypomagnesemia [145]. It is known to prolong QT interval and may lead to ECG abnormalities and arrhythmias therefore caution is advised when prescribing in cardiac patients [154]. Likewise, clinicians should keep in mind drug interactions and concomitant use of posaconazole with rifabutin, phenytoin, ritonavir, fosamprenavir, benzodiazepines, calcium channel blockers, digoxin, cimetidine, and ergot alkaloids should be best avoided [153].

*Efficacy in mucormycosis:*

Posaconazole as an extended spectrum anti-fungal has demonstrated a better efficacy against mucormycosis as compared to itraconazole. It is used in three different categories for the treatment of mucormycosis: (i) therapeutic, where it is given as a first line agent or where amphotericin B is not tolerated, (ii) prophylactic, in cases which are prone to develop mucormycosis e.g., transplant patients, HIV patients etc., and (iii) as salvage therapy for de-escalated, refractory and resistant cases [151].

In-vitro studies for mucormycosis reveal that posaconazole appears slightly better than itraconazole and significantly better than voriconazole and fluconazole. It has also shown survival benefit in animal models [155]. Greenberg et al. have shown clinical success in 83% of the cases who were treated for mucormycosis [142]. Another study by Manesh et al. demonstrated efficacy of posaconazole in ROCM and their results showed complete cure in 66.6% of cases and partial response in 16.6% cases. Their study concluded that posaconazole should be considered a safe and effective alternative for ROCM cases especially if toxicity prevents use of amphotericin B [156].

It has been depicted that at doses of 200 mg TDS, posaconazole is significantly more effective in treating breakthrough invasive fungal infections as compared to fluconazole [157]. So far, large multicenter trials have not been conducted therefore efficacy cannot be determined with certainty. A combination of tacrolimus and posaconazole was tested in an in-vitro and in-vivo study by Lewis et al. and their results showed that the combination results in synergism with better results than monotherapy with posaconazole [158].

To this point, posaconazole is used as first line agent in cases that are resistant or intolerant to amphotericin B or as a salvage option. It is also recommended as drug of choice for prophylaxis against COVID associated mucormycosis in susceptible individuals [118,159]. Posaconazole has also been used in conjunction with amphotericin B and this combination allows reduction in the overall dose of both drugs leading to better tolerability and efficacy [160]. The use of posaconazole in ROCM cases has been shown to prevent the need for orbital exenteration [161].

The success rate of posaconazole salvage therapy as stated by Sipsas et al. was around 70% when the drug was administered at the dose of 200 mg QID. In another study, 61% patients showed complete a response and 21% showed a partial response when posaconazole oral suspension was used for four months [159].

**D. Isavuconazole:**

Isavuconazole is a newly developed second generation, extended spectrum. It is water soluble triazole antifungal drug, which has been showing promising results in various yeast and mold infections. Isavuconazole is available as a water-soluble pro-drug, isavuconazonium, which is a tetrazolium salt linked to an aminocarboxyl chain. Its spectrum of activity is broader than other similar azoles and is available for oral and IV use. Depending on the target species and the dosing, isavuconazole can be fungistatic or fungicidal [162,163].

*Mechanism of Action:*

Similar to other azoles, isavuconazole disrupts the fungal cell membrane by inhibiting the CYP-450 dependent synthesis of ergosterol through lanosterol 14-demethylase. Fungal cell death follows this disruption. The structure of isavuconazole contains a side arm, which has affinity for fungal CYP51 protein leading to a broader spectrum of fungicidal activity [162,164].

*Dosing and Administration:*

Isavuconazole is available as oral (100 and 200 mg) and IV formulations. For IV drug, unlike voriconazole, addition of cyclodextrin is not needed to achieve solubility which eliminates cyclodextrin related renal toxicity making it a safer alternative to voriconazole. Peak plasma levels are achieved in 1.5 to 3 h after administration and half-life of the drug has been found to be around 56 to 77 h. After metabolism through plasma esterases, it is converted to the inactive por-drug, isavuconazonium [162,165].

The recommended doses of isavuconazole for treating mucormycosis are 200 mg twice daily on day one and two followed by 200 mg daily for 1 to 2 weeks or until the infection improves [166].

Isavuconazole has remarkable bioavailability and predictable pharmacokinetics. The absorption is independent of food and the drug, and is water soluble. Absence of cyclodextrin make it relatively liver friendly [162].

*Spectrum of activity:*

Various in-vitro, animal based in-vivo; phase III human based clinical trials have been conducted, which have revealed the spectrum of activity to include Candida species, *cryptococcus, geotrichum, tricosporon, saccharomyces*, invasive fungal infections caused by *aspergillus* species, *lichtheimia* (absidia), mucor species, *Rhizopus, fusarium, trichophyton*, and *syncephalastrum* species [162].

Clinical research has shown that the efficacy of isavuconazole is similar to that of posaconazole and Voriconazole for treating oro-pharyngeal candidiasis. It has also depicted activity against fluconazole and amphotericin B resistant Candida infections. In-vitro and animal-based studies have proven the effectiveness of isavuconazole for successfully clearing infection caused by invasive molds like *zygomycetes* and *aspergillus*. Similarly, it has been shown to clear disseminated disease caused by these organisms. In-vitro studies have shown that isavuconazole is effective against *aspergillus terreus*, which in most cases is resistant to amphotericin B. in addition to this, isavuconazole have been found to depict in-vitro activity against itraconazole and voriconazole resistant species of *aspergillus lentulus* [163].

Isavuconazole can be used for fungal infections of the brain, but clinical data is scarce, and no definite conclusion can be drawn from the cases reported in literature.

*Adverse effects and contraindications:*

In general. Isavuconazole enjoys good tolerability with minimal adverse effects. Commonly reported side effects include nausea, vomiting, and diarrhea, which normally do not require treatment discontinuation. Hepatotoxicity is a known side effect for all antifungal medications and the same is true for isavuconazole therefore monitoring of liver enzymes is recommended. Contrary to posaconazole, it shortens the QT interval, and this shortening is directly related to the dose of the drug being administered. Other uncommon side effects can be skin rashes, pedal edema, muscle pain, insomnia, and a fall in blood pressure.

Since isavuconazole is an inducer of the hepatic enzyme, co-administration with ketoconazole, rifampin, carbamazepine, ritonavir, barbiturates, sirolimus, tacrolimus, cyclosporine, colchicine, and digoxin should be avoided at all costs [162,164].

*Efficacy in mucormycosis:*

In the US and EU, isavuconazole is approved for the treatment of invasive mucormycosis. Single arm Phase III VITAL trails has shown isavuconazole is effective against mucormycosis and the activity is not inferior to amphotericin B, which till date is considered the gold standard of treatment care. Itraconazole and voriconazole have limited activity against mucormycosis and isavuconazole has shown superior efficacy in comparison to these azoles [165]. Graves et al. has demonstrated isavuconazole activity against mucormycosis to be better than posaconazole when used as salvage therapy for invasive pulmonary infection [166]. To this date no randomized control trials have been done therefore it is not possible to draw a definite conclusion and most of the data available is in the form of case reports and case series. Isavuconazole can be administered as prophylactic treatment for susceptible cases but can lead to breakthrough fungal infections just like the case with voriconazole and posaconazole [164]. Isavuconazole has also been used as salvage therapy for treating invasive mucormycosis in pediatric patients and satisfactory results have been demonstrated in the case series by Ashkenazi-Hoffnung et al. [167]. Marty et al. reported a mortality of 33% in their series when they treated mucormycosis with isavuconazole versus 39% for amphotericin B with the conclusion that the efficacy of isavuconazole is similar to amphotericin B for invasive mucormycotic infections [168]. Miceli et al. have reported an overall response rate of 31.4% for his cases which included both the categories; primary infection and salvage cases. They report mortality rate to be higher in refractory cases (43%) as compared to primary infection cases (33.3%) [162].

Keeping all this data and figures under consideration, it can be assumed that isavuconazole can be an effective alternative to amphotericin B and can be considered as a primary treatment option for salvage therapy in amphotericin intolerant or resistant cases.

**E. Echinocandins**

Echinocandins comprise a group of semi-synthetic anti-fungal drugs, which contain an N-linked acyl lipid side chain of cyclic lipopolypeptides. The three approved drugs included in this category are caspofungin, micafungin, and anidulafungin [169].

*Mechanism of Action:*

The mechanism of fungal killing is unique for echinocandins. They non-competitively inhibit the enzyme formation of 1,3-β-D-glucan which is a principal component in the wall of the fungal cell and is employed in production of glucan. As a result, the fungal cell walls are damaged leading to anti-fungal action [170,171].

*Dosing and Administration:*

Echinocandins demonstrate poor bioavailability when administered orally therefore they are not marketed in oral formulation and the only available form is IV [172]. The vials are available as either 50 mg/vial or 70 mg/vial. For Candida and *aspergillus* infections, usually a loading dose of 70 mg is given on day 1 followed by 50 mg for the next 14 days; however, it can be continued depending on the response and severity of the disease [173].

*Spectrum of activity:*

As the general description goes, echinocandins are fungicidal for Candida and fungistatic for invasive fungal infections caused by *aspergillus spp.* Echinocandins have been found to be efficacious against amphotericin B and fluconazole resistant Candida [174]. Candida species, such as *C. parasilosis* and *C. guilliermondii*, are however found to be resistant to echinocandins [175]. Similarly, virtually no activity is demonstrated against fungi lacking β-glucan in their cell wall, which is the primary target agent for echinocandins. These include cryptococcus neoformans, trichosporon, and zygomycetes [176,177].

*Adverse effects and contraindications:*

The commonly encountered adverse effects are infusion related and include facial edema, swelling, rash, itching, and fever. They are usually not life threatening and generally do not require discontinuation of the therapy, which can be minimized using anti-histamines. As with other antifungals, echinocandins adversely affect liver function and monitoring of LFTs is recommended during the course of the treatment especially in case of caspofungin. Dose should be reduced to half (35 mg) in mild to moderate hepatic insufficiency. It can be administered safely in renally deranged patients. Echinocandins are generally not prescribed in children [178].

*Efficacy in mucormycosis:*

It has been demonstrated through animal and human studies that echinocandins possess limited activity against zygomycetes and mucormycosis [179]; however, caspofungin shows some activity against *Rhizopus oryzae* because of presence of a target enzyme. It has been expressed that caspofungin can be used in combination with amphotericin B for a synergistic effect especially in cases of disseminated disease caused by R oryzae [180]. The authors showed 50% improvement in survival when then they combined caspofungin and liposomal amphotericin B as compared to amphotericin B alone [181]. The same effect has been shown with combining amphotericin B and micafungin and anidulafungin. However, significant differences in the efficacy among the three different echinocandins have yet to be proven [182].

A study involving a small number of patients presenting with ROCM showed significantly improved survival when caspofungin was combined with amphotericin B as compared with amphotericin B alone [183]. Further multicenter studies with a larger number of patients are needed to reach a definitive conclusion.

**F. Iron Chelators (Deferoxamine and Deferasirox)**

Deferoxamine and deferasirox belong to a group of medicines referred to as chelators. They are primarily used in iron and aluminum toxicity and is approved by FDA to treat iron overload in patients requiring frequent transfusion like those of sickle cell disease or thalassemia [184]. Molecularly deferoxamine is a hexadentate, produced by fermentation of Streptomyces piloses and possesses the ability to bind iron in a ratio of 1:1. Deferasirox is a newer generation of iron chelators, which possess twice the affinity as demonstrated by deferoxamine and can bind iron in a ratio of 2:1. In hemolytic and thalassemic patients, deferasirox demonstrates higher efficacy as compared to deferoxamine [185].

*Mechanism of Action:*

Deferoxamine binds and chelates free iron (non-transferrin bound), hemosiderin and ferritin. After binding the drug-iron complex is formed known as ferrioxamine, a water-soluble compound, which is then excreted either through bile or urine [186].

*Dosing and Administration:*

Oral absorption of deferoxamine is poor, hence it has to be given parenterally. The routes available are IM, subcutaneous (SC), IV with SC being the most favored route. A 25 G butterfly needle is used for this purpose and abdomen is usually the favored area for administration. 10% solution of deferoxamine (40–60 mg/kg/day) is infused SC route over 10 to 12 h through an infusion pump for 4- to 5-days per week. Co-administration of vitamin C along with deferoxamine enhances its therapeutic efficiency as vitamin C increases the concentration of chelatable iron but caution is advised as this can lead to iron toxicity hence clinician needs to balance both issues.

Deferasirox is available in oral suspension, tablet, and granule form and as IV formulations. Initial dose is 20 mg/kg/day with a maximum dose of 40 mg/kg/day. Creatinine clearance must be monitored while administering deferasirox [187].

*Adverse effects and contraindications:*

Sensorineural deafness and vision loss are known adverse effects of long term deferoxamine therapy. The effects are reversible only if the therapy is discontinued as soon as they start to appear. In addition to deafness and blindness, other reported side effects include GI disturbance, erythematous lesions on the skin and anaphylactic reactions. Nausea, vomiting, gastric discomfort increased serum creatinine, and proteinuria are commonly reported side effects for deferasirox.

Overall tolerability of the drug is high however the use is contraindicated in known hypersensitivity cases and in those who have renal disease. Caution is advised when the clinician wishes to prescribe during pregnancy (category C drug) [185,188].

*Efficacy in mucormycosis:*

Mucorales species require free iron for growth and iron chelators, such as deferoxamine and deferasirox, possess antifungal properties by producing iron starvation. Therefore, it can be combined with amphotericin B, but renal function must be monitored closely as both have the potential of producing nephrotoxicity. Potential benefit has been demonstrated using triple therapy with amphotericin B, echinocandins and deferoxamine. Deferoxamine has been reported to promote fungal infection because it increases the free iron in the body, but this effect has not been seen with deferasirox because of its higher affinity for free iron. The combination of deferasirox with liposomal amphotericin B has also been used as salvage therapy with promising results [58,68,189]. However, the possible utility of adjunctive deferasirox has only been evaluated in small studies with mixed results. In addition, deferasirox as an adjunctive agent for mucormycosis has not been adequately studied in humans and therefore, it should not be used.

##### Topical Agents

Generally, there is limited role of topical treatment in deep invasive fungal infections; however, Kontoyiannis et al. have stated that wounds of the patients afflicted with mucormycotic infections can be washed with amphotericin B solution and dressings can be soaked with amphotericin B before packing inside the defect cavities. This increases the contact time between the drug and the diseases site thus enhancing efficacy of the treatment [118].

#### 5.2.2. Surgical Treatment

Surgical debridement of Mucormycotic lesions is considered to be the mainstay and must be initiated as early as possible during the course of the treatment. Repeated and aggressive debridement sessions are at times needed to improve survival and to reduce chances of disease spread [118].

The surgical treatment of ROCM ranges from turbinectomy to the point of aggressive removal of orbital contents and neurosurgical debridement when the disease has involved the cranial cavity [190]. The extent of surgery is usually guided by the extent of the disease, which is assessed both clinically as well as radiologically using CT scans and/or the MRIs [130]. A timely and adequately performed surgery significantly improves survival of such patients; therefore, any patient who is deemed medically fit to undergo surgery should be operated upon without any delay [116]. The surgery for ROCM usually involves a team approach between otorhinolaryngologists, ophthalmologists, oral and maxillofacial surgeons, plastic surgeons, and neurosurgeons [191].

##### Turbinectomy

Nasal cavity is usually considered as the portal of entry for the fungus; therefore, involvement of nasal turbinates is a common occurrence. From the nasal mucosa, the fungal hyphae move on to involve adjacent structures [192]. The presence of black colored turbinates’ on clinical or endoscopic examination and a non-enhancing turbinate on an MRI (referred to as black-turbinate sign) is an important finding in involvement of turbinates’ by mucormycosis [193]. Turbinectomy is a surgical procedure which involves removal of some or all of the turbinates’, which are small bony structures found inside the nose and are commonly three to four in number [194].

Turbinectomy is usually carried out to relieve chronic nasal congestion, nasal bleeding or to treat sleep apnea and a deviated nasal septum. The surgical approach to remove the inferior turbinate is usually through the nostrils and can be conducted through electrocautery, microdebrider, radiofrequency or an endoscope. In case of mucormycosis, the middle meatus, lateral nasal wall, or the ethmoidal sinuses may have to be debrided along with removal of the turbinates [193].

Debridement of the Maxillary, Ethmoidal and Frontal Sinuses

Once the fungus has gained entry into the nasal cavity, its further course is dictated by the immune status of the host. In most of the immunocompromised hosts, the next target is the maxillary sinuses. Involvement of sinuses present with facial pain, paresthesia of the infraorbital nerve, headache, nasal blockage, nasal bleeding, and/or dental pain [192]. The CT scan generally shows dense and non-homogenous mass along the nasal cavity with hyperdense opacified masses in the maxillary and/or ethmoidal sinuses. There can be presence of dead necrotic bone fragments along the anterior, inferior or the posterior walls of the maxillary sinuses [195].

Once involved, the sinuses require extensive debridement, which can be carried out intraorally through the Caldwell Luc approach, endoscopically using FESS or through the lateral rhinotomy approach [196,197]. When the disease spreads to involve the frontal sinus, the debridement usually requires a Lynch–Howarth incision or the endoscopic approach which prevents the external scarring and disfigurement associated with Lynch–Howarth incision [198,199].

The common complications include numbness of the cheek and upper alveolus area, damage to the nasolacrimal duct, sagging of the cheek area if the anterior maxillary wall is removed, recurrence of infection, damage to the roots of the teeth, and facial scarring if maxillary sinus debridement is performed through an open approach [200]. Ethmoidectomy can lead to intraorbital hemorrhage due to damage to anterior ethmoidal artery. Damage to extraocular muscles leading to ophthalmoplegia has also been reported after sinus surgery [201].

##### Maxillectomy/Palatal Resection

If the disease spreads from the maxillary sinus towards the oral cavity, it often causes angioinvasion of the palatal blood vessels leading to thrombosed necrotic black colored palate. The necrosis usually spreads towards the alveolus leading to destruction of the bone around the teeth causing tooth mobility and loss. Anterior and posterior spread from the sinus mucosa leads to destruction of the anterior and posterior wall of the maxilla, respectively. Involving these walls requires surgical debridement and removal, the extent of which is guided by the involvement of the bone by the fungus [192].

Depending on the involvement and formation of bony sequestrum, the resection can be inferior/partial maxillectomy, sub-total maxillectomy with sparing of the orbital floor or total maxillectomy with resection of the orbital floor as well. Likewise, if the disease is involving both sides of the jaws, the patient may require bilateral debridement, which can be accomplished in a single setting or in multiple sessions. The approach for maxillary and palatal debridement can be either intraoral using a vestibular incision in the buccal sulcus or extra-oral using a Weber–Fergusson incision [190,202]. Use of a frozen section is recommended in order to ensure complete removal of the dead and devitalized tissue if planning for resection in a single setting [203].

Whenever the maxilla along with palate is resected, there is a resultant defect in the oral cavity leading to formation of an oro-nasal/oro-antral communication, which adversely affects the patient’s quality of life in terms of mastication and phonation and therefore requires reconstruction in order to restore the normal functioning of the stomatognathic system. The reconstruction of the maxillary defects can be conducted through the fabrication of obturators, which can be tooth/mucosa supported or implant supported [204,205]. Zygomatic implants have emerged as a successful entity in such cases [206]. Various authors have reported use of osteo-cutaneous free flaps like free fibula flap, radial forearm, and deep circumflex iliac artery flap with successful uptake [203,207].

##### Orbital Exenteration

Orbital contents get involved by mucormycosis when the disease spreads superiorly from the maxillary sinus. The other pathway that explains the orbital involvement is through the pterygopalatine and infratemporal fossa. The orbital involvement should be suspected if the examination of the eye reveals chemosis and eyelid oedema, corneal numbness, ophthalmoplegia, pupillary abnormalities, nystagmus and/or loss of visual acuity. Superior orbital fissure syndrome due to thrombosis of superior ophthalmic vein and orbital apex syndrome can also be presenting signs and symptoms depicting severe orbital involvement by the fungus and are considered sinister signs and it is generally not possible to save a patient’s eye once they appear [208,209,210].

Orbital exenteration is a procedure involving resection of the whole of the contents of the orbit, which includes periorbita, eyelids with or without the adjoining skin. The surgery is potentially disfiguring and is reserved for cases which show positive findings in the eyes and are not responsive to the medical management [211,212].

The resultant defect can be reconstructed using split or full thickness skin graft, pedicled myocutaneous flaps utilizing latissimus dorsi, the pectoralis major flap, and the temporalis muscle flap [212,213]. Reconstruction using free flap usually utilizes rectus abdominus flap, radial forearm free tissue transfer and anterolateral thigh flap [214].

### 5.3. Complications

Complications can be experienced from the disease itself or from the treatment of that disease.

#### 5.3.1. Complications of the Disease

Mucormycosis originating maxillary sinuses in a diabetic patient mostly spreads to the rhino-cerebral region whereas patients having a healthy immune status would have mucor limited to skin or involving the lungs. If there is ROCM, it would involve the paranasal sinuses and maxillary sinus extending to the palate, skin of the cheek and nose area, orbit, brain, and the cranial bones [192]. Angioinvasion is the hallmark of mucormycosis, and formation of intravascular thrombi leads to infarction and necrosis of the involved tissue. When the disease progresses to the cranial cavity, there can be brain infarction or intracranial hemorrhage due to damage to the vessel walls causing a cerebrovascular accident which can be fatal for the patient [203]. Formation of brain abscesses, skull base erosions and cavernous sinus thrombosis has also been reported as a complication of mucormycosis. Similarly, extension to the disease involving meninges can lead to development of fungal meningitis [202].

Extension of the disease to involve the orbital contents can present with ophthalmoplegia and decreased visual acuity. Complete blindness can ensue if the central artery of retina gets thrombosed. Ophthalmoplegia is secondary to involvement of cranial nerves III, IV and/or VI. Orbital cellulitis, conjunctival hemorrhage and orbital edema are other signs of orbital involvement by the fungal hyphae. Progressive involvement of the facial and trigeminal nerve is also a frequent finding with most of the patients complaining of numbness in the area of the cheek and the side of the nose [215]. Involvement of the facial/nasal skin, maxillary alveolus, palate, nasal bridge, ethmoidal, sphenoidal, and frontal sinuses have also been reported in the literature [216].

The disease can extend posteriorly from the maxillary sinuses and nasal cavity to involve the infratemporal fossa, pterygopalatine fossa and muscles of mastication leading to development of trismus and decreased mouth opening. From the infratemporal and pterygopalatine fossa disease can easily spread to involve the cranial base and the orbits [192].

#### 5.3.2. Complications of Medical Treatment

Nausea, vomiting, diarrhea, GI disturbances, hepatotoxicity, nephrotoxicity, cardiotoxicity, infusion related complications, facial swelling, rash, sensorineural deafness, loss of color vision, headache, fever, loss of appetite, weight loss, electrolyte disturbances, and development of breakthrough fungal infections are some of the reported complications of the various medications used for the treatment of ROCM [217].

#### 5.3.3. Complications of Surgical Treatment

Post-operative complications include difficulty in eating, speaking, and nasal regurgitation of fluids if there is a palatal defect. If the resection includes orbital exenteration then blindness would be a post-operative complication too. Difficulty in oral functioning often leads to an overall low quality of life. Facial, nasal, and oral deformities are known complications of the surgical management of ROCM [218]. These resultant defects require reconstruction in the form of loco-regional or distant flaps with or without prosthesis for dental and palatal defects [207].

### 5.4. Prognosis

Prognosis of any disease largely depends upon the extent of spread, the aggressiveness of disease and the time of treatment. The immune status of the patient, particularly the presence of any hematologic disease, plays an important role. Localized infections in the case of mucormycosis have a better prognosis while intracranial extension is a poor prognostic indicator. Studies have shown that rhino-cerebral disease in absence of any systemic illness has 75% survival rate. The survival rate reduces to 20% if there is a systemic disease along with ROCM [219]. Mucor is considered to have a high mortality rate if orbito-cerebral involvement is seen with pulmonary disease. The prognosis is much better if the organism has not invaded beyond the sinus before the surgical intervention [220].

Another important prognostic factor is the time at which the disease was diagnosed and treated. Pagano et al. reported mostly patients of mucormycosis died within 12-weeks’ time of diagnosis, whereas aspergillosis has a better survival outcome [221]. Chamilos et al. reported mortality rate to jump from 48.6 to 82.9% by delaying the amphotericin B base therapy for more than 5 days, especially in patients with hematologic malignancies [78]. If mucormycosis affected patients receive surgical and medical treatment on time, there are chances of mortality rate falling down to less than 20% [68]. ROCM can be diagnosed much earlier than pulmonary mucormycosis thus ROCM has a better chance of survival with early diagnosis and treatment. On the other hand, pulmonary mucormycosis has a mortality rate up to 65% as it is difficult to diagnose and mostly occurs in neutropenic patients [222]. Overall reported mortality with all forms of mucormycosis range from 40–80% with survival rates significantly worse in patients with hematological malignancies and organ transplantation. Similarly, neonatal patients afflicted with mucormycosis show poor prognosis [223]. Cerebral involvement in an immunocompromised patient usually ends up in fatality. The survival in a rapidly progressive disease is reported to be 3 to 6 weeks [203], A follow-up period of at least 36 months is recommended as there is a 13% possibility of reoccurrence of disease during the 3-year post-operative period [224]. At 5-year follow-up, an overall survival of around 60% has been reported [225].

### 5.5. Future Directions/Recommendations

Initial signs and symptoms of ROCM may be subtle and patient may present initially with nonspecific pain in the maxilla or cheek area or may present with a loose tooth in the maxilla, which can easily be mistaken for an odontogenic etiology and thus liable to incorrect diagnosis and inappropriate treatment. Dentists, oral and maxillofacial surgeons, otorhinolaryngologists, may be first responders in cases of previously undiagnosed ROCM and if they are not vigilant and cautious, they can miss the diagnosis till it’s very late. It is therefore suggested that thorough history should be obtained and detailed clinical examination should be conducted in patients who present with non-specific pain/pus discharge in the maxillary/facial region or those who have non healing extraction sockets/OAFs after extraction/osteomyelitis [226].

The clinicians should be aware of the red flags such as previous history of COVID-19, especially those who were hospitalized and given high flow oxygen therapy along with high dose long term steroids; metabolic diseases, such as diabetes especially if uncontrolled; patients taking immunosuppressive treatment for any reason; or patients who may be immunosuppressed in any other way [227]. The case must be investigated thoroughly if any of the clinical presenting signs of ROCM are present in a pre-disposed individual followed by aggressive surgical management supported medically. It is better to err on the side of safety rather than waiting and watching. Similarly, it is the job of dentists and dental hygienists to inform pre-disposed individuals about the risks of developing ROCM and importance of maintaining optimal oral hygiene. Likewise, otorhinolaryngologists should counsel these patients about maintaining appropriate nasal hygiene. Physicians treating COVID-19 patients should use steroids judiciously so that incidence of post-COVID mucormycosis can be minimized as much as possible.

## 6. Conclusions

There was an increased surge in the incidence of mucormycosis during the second wave of the COVID-19 pandemic across the globe. This review has presented the clinical features of mucormycosis generally and its association with COVID-19, which requires a multidisciplinary management approach along with radiological correlation, and pharmacological and surgical management of mucormycosis involving the oral and dental region. ROCM is an invasive, opportunistic fungal infection that is progressive and has a high mortality rate. Patients with uncontrolled diabetes mellitus, predisposing co-morbidities, and immunocompromised are at higher risk if not treated at an appropriate time. Therefore, it is imperative to predict and treat the causes of ROCM to achieve a favorable outcome if diagnosed at the earlier stage, resulting in a better prognosis if the organism has not invaded beyond the sinus before the surgical intervention. There are higher chances of survival if ROCM can be diagnosed early and receive surgical and medical treatment on time.

## Figures and Tables

**Table 1:** Critical predisposing factors which increase mucormycosis vulnerability.

Blood Associated Malignancies (Lymphoma, Leukemia and Myeloproliferative Disorders)
Uncontrolled diabetes mellitus concurrent with ketoacidosis
High dose corticosteroids/immuno-suppressive drugs for 2–3 weeks
Solid organ malignancies
Solid organ transplantation
Therapy with Deferoxamine
Metabolic acidosis
Hematopoietic stem cell transplantation
Rheumatologic disorders
Multiple transfusions
Neonatal prematurity
Malnutrition
Prophylaxis with voriconazole (breakthrough invasive fungal infections)

**Table 2:** Factors increasing mucormycosis vulnerability in immunocompetent individuals.

Skin injuries, burns, trauma
Contaminated bandages, tongue depressors.
Combat-related injuries
Intravenous drug abuse
Prolonged hospital stays

**Table 3:** Oral and dental manifestations of rhino-orbital-cerebral mucormycosis [114].

Dental pain
Mobile teeth
Halitosis (bad breath)
Nasal stuffiness
Nasal discharge with epistaxis, black purulent discharge
Necrotic bone/Sequestrum formation in the palate and maxillary alveolus Formation of Oro-antral/Oro-nasal communications/fistulae Non-healing extraction sockets with signs similar to alveolar osteitis or chronic osteomyelitis. Trismus due to involvement of muscles of mastication Para-sinusal pain
Intraoral/Extra oral draining sinuses
Erythema of nasal mucosa
Palatal ulceration
Facial erythema
Black discoloration of skin
Periorbital erythema and edema, cellulitis
Orbital Pain, Ptosis, Diplopia, Vision loss, Ophthalmoplegia Headache

**Table 4:** Summary of medicinal management of mucormycosis.

Drug Name	Class of Drug	Mechanism of Action	Administration	Dosage	Side Effects/Contraindications/Warnings	Role in Mucormycosis
Amphotericin B	Polyene	Damage to fungal cell by binding to ergosterol	IV	Dose of Amphotericin B deoxycholate is 1–1.5 mg/kg/day while dose of Liposomal Amphotericin B is 5–15 mg/kg/day [68].	Electrolyte disturbances Nephrotoxicity, hepatotoxicity, neurotoxicity	1st line agent in all cases unless contraindicated or not tolerated by the patient.
Itraconazole	Azole	Inhibition conversion of lansosterol to ergosterol by blocking 14-α-demethylase	Capsules, oral solution and IV	100–200 mg/day	GI disturbances Hypertension Cardiotoxicity	Minimal activity. 2nd or 3rd line agent where better azoles are not available and amphotericin B cannot be used.
posaconazole	Azole	Inhibition conversion of lansosterol to ergosterol by blocking 14-α-demethylase	Oral suspension, delayed release tablet and IV	200–300 mg/day	GI disturbances Hepatotoxicity Nephrotoxicity Infusion related reactions	Used as prophylactic agent in mucormycosis prone individuals 2nd line agent after amphotericin B.Useful as salvage therapy.
Isavuconazole	Azole	Inhibition conversion of lansosterol to ergosterol by blocking 14-α-demethylase	Oral and IV	200 mg/day	GI disturbances Hepatotoxicity Prolongs QT interval Skin rashes	So far, the best azole with efficacy comparable to amphotericin B and can be used as first line agent. Useful as salvage therapy.
Echinocandins	Cell wall inhibitor	Inhibits enzyme 1,3-β-D-glucan causing damage to fungal cell wall	IV	50–70 mg/day	Infusion related reactions Hepatotoxicity	Used as combination therapy with amphotericin B.
Deferasirox	Chelators	Chelates and removes excess iron	IV	40–60 mg/day	Sensorineural deafness Blindness Skin eruptions Anaphylactic reactions	Used in combination with amphotericin B for reducing iron overload. Considered adjunctive treatment in mucormycosis.
